# Trends in osteoporosis care patterns during the COVID-19 pandemic in Alberta, Canada

**DOI:** 10.1007/s11657-022-01132-7

**Published:** 2022-08-03

**Authors:** T. Oliveira, J. Brown, A. G. Juby, P. Schneider, R. J. Wani, M. Packalen, S. Avcil, S. Li, M. Farris, E. Graves, S. McMullen, D. L. Kendler

**Affiliations:** 1 Amgen Canada Inc, Mississauga, ON Canada; 2 Medlior Health Outcomes Research Ltd, Calgary, AB Canada; 3 Department of Medicine, Division of Rheumatology, Laval University and CHU de Québec Research Centre, Quebec City, QC Canada; 4 Department of Medicine, Division of Geriatric Medicine, University of Alberta, Edmonton, AB Canada; 5 Division of Orthopaedic Trauma, Department of Surgery, University of Calgary, Calgary, AB Canada; 6 Department of Medicine, Division of Endocrinology, University of British Columbia, Vancouver, BC Canada

**Keywords:** COVID-19, Pandemic, Osteoporosis, Care patterns, Healthcare resource utilization

## Abstract

**Purpose/introduction:**

The objective of this study was to describe osteoporosis-related care patterns during the coronavirus disease 2019 (COVID-19) pandemic in Alberta, Canada, relative to the 3-year preceding.

**Methods:**

A repeated cross-sectional study design encompassing 3-month periods of continuous administrative health data between March 15, 2017, and September 14, 2020, described osteoporosis-related healthcare resource utilization (HCRU) and treatment patterns. Outcomes included patients with osteoporosis-related healthcare encounters, physician visits, diagnostic and laboratory test volumes, and treatment initiations and disruptions. The percent change between outcomes was calculated, averaged across the control periods (2017–2019), relative to the COVID-19 periods (2020).

**Results:**

Relative to the average control March to June period, all HCRU declined during the corresponding COVID-19 period. There was a reduction of 14% in patients with osteoporosis healthcare encounters, 13% in general practitioner visits, 9% in specialist practitioner visits, 47% in bone mineral density tests, and 13% in vitamin D tests. Treatment initiations declined 43%, 26%, and 35% for oral bisphosphonates, intravenous bisphosphonates, and denosumab, respectively. Slight increases were observed in the proportion of patients with treatment disruptions. In the subsequent June to September period, HCRU either returned to or surpassed pre-pandemic levels, when including telehealth visits accounting for 33–45% of healthcare encounters during the COVID periods. Oral bisphosphonate treatment initiations remained lower than pre-pandemic levels.

**Conclusions:**

This study demonstrates the COVID-19 pandemic and corresponding public health lockdowns further heightened the “crisis” around the known gap in osteoporosis care and altered the provision of care (e.g., use of telehealth and initiation of treatment).

**Summary:**

Osteoporosis has a known substantial care and management disparity, which has been classified as a crisis. The COVID-19 pandemic created additional burden on osteoporosis patient care with healthcare encounters, physician visits, diagnostic and laboratory tests, and treatment initiations all declining during the initial pandemic period, relative to previous years.

**Supplementary Information:**

The online version contains supplementary material available at 10.1007/s11657-022-01132-7.

## Introduction

Osteoporosis is one of the most common chronic diseases, afflicting over 200 million people worldwide [1]. According to the International Osteoporosis Foundation, among those aged at least 50 years, 1 in 3 women and 1 in 5 men will experience an osteoporotic fracture in their lifetime [2–5]. Worldwide, osteoporosis is estimated to cause 8.9 million fractures annually; of which, hip, forearm, and vertebral fractures are the most common fracture sites [6]. In Canada, the prevalence of osteoporosis is projected to increase in the coming years with a growing and aging population [7]. According to the Public Health Agency of Canada (PHAC), fractures associated with osteoporosis are a main public health concern causing significant morbidity, mortality, and costs [7].

Although osteoporosis is a prevalent disease, there has been a substantial care and management disparity, which has been classified as a crisis [8, 9]. The Fragility Fracture Network initiated a global call to action to improve care of people presenting with low-energy fractures [10]. According to the PHAC 2020 report focusing on trends in incidence of osteoporosis diagnoses and fracture rates between 2000/01 and 2015/16, osteoporosis diagnoses decreased over time, while fracture rates remained stable [7]. Furthermore, among those who had an osteoporosis-related fracture, less than 20% of individuals received an osteoporosis diagnosis, bone mineral density (BMD) test, or an osteoporosis-related medication prescription within 1 year, despite effective therapy being widely available [7]. This evidence suggests that patients and care providers are not aware or taking appropriate action to deal with the disease underlying their fracture, i.e., osteoporosis, and this has the potential to lead to under management of patients with low-energy fractures.

Practitioners have been urged to focus on improving secondary fracture prevention, since these patients are at highest risk for a future fracture. In Canada, only the provinces of Alberta and Ontario have formal government-supported fracture prevention action plans and combined, contain 83% of Canada’s fracture liaison services despite only having 50% of the country’s population [11, 12]. In a Canadian study from Ontario focusing on the primary versus secondary fracture prevention gap, among patients who were identified as potentially at elevated risk for fracture, 62% did not complete BMD testing. The most common reasons for not receiving a BMD test were that physicians intended to order a BMD test at a later date, physicians did not think BMD testing was necessary, or the patient refused [13]. Furthermore, among those patients with BMD tests, only 29% had completed 10-year fracture risk scores, and for patients with high fracture risk scores, 37% did not receive clinical guideline recommended osteoporosis medications [13]. Furthermore, a real-world evidence study conducted in five European countries prior to the coronavirus disease 2019 (COVID-19) pandemic examined osteoporosis medication patterns among new users [14]. They observed oral bisphosphonates and denosumab as the most common treatments prescribed for patients with osteoporosis, and a large proportion of patients stop treatment without starting any other new treatment thereafter [14], further providing evidence of insufficient ongoing osteoporosis care and management.

With significant strains to the healthcare system in 2020 due to the COVID-19 pandemic, it was anticipated that osteoporosis care would be deprioritized, thus creating an even wider gap in care and management. A retrospective analysis of global google analytics data conducted between February and April 2020 found that compared to February 2020 numbers, the number of daily sessions on the FRAX® website for online fracture risk assessments fell 23.1% on March 2020 and 58.3% on April 2020 [15]. Furthermore, a global survey of healthcare workers from 53 countries focusing on the impacts of the COVID-19 pandemic on osteoporosis care reported an increase in telemedicine consultations, and delays or interruptions in care and management, including BMD testing, supply of osteoporosis medications, and parenteral medication delivery [16]. Specifically, 43% of clinicians reported difficulty in administering appropriate medication and only 29% of patients were able to obtain a dual-energy x-ray absorptiometry (DXA) scan for BMD testing as recommended [16]. Prior to the COVID-19 pandemic, alendronate, one of the most prescribed oral bisphosphonates for osteoporosis, reported increased prescription uptake by new users in 2019 in Europe. However, after the COVID-19 pandemic lockdowns started on March 2020, rates of new use of alendronate rapidly decreased, with only some recovery to pre-pandemic levels after the initial lockdown (June to September 2020) [17]. Although limited, initial evidence has shown that osteoporosis care and management may have been deprioritized because of the COVID-19 pandemic priorities and response. Additional research, particularly from a Canadian population-based perspective, will help to understand the impact of the COVID-19 pandemic on osteoporosis care and management in a Canadian setting.

The objective of this study was to describe changes in osteoporosis-related care patterns including patients with an osteoporosis-related healthcare encounter, physician visits, diagnostic and laboratory test volumes, and treatment prescription dispense patterns during the COVID-19 pandemic in Alberta, Canada, relative to the three-years preceding.

## Methods

### Study design

This study used a repeated cross-sectional study design, using three-month periods of population-level administrative health data from the entire province of Alberta, Canada, between March 15, 2017, and September 14, 2020 (Supplementary Fig. 1).

### Data sources and outcomes of interest

The study population represents Alberta residents aged ≥ 50 years with osteoporosis-related healthcare resource utilization (HCRU) and treatment utilization during each cross-sectional period. Outcomes of interest included patients with osteoporosis-related healthcare encounters and resource utilization, including physician visits, hospital- and ambulatory care-based BMD diagnostic tests, vitamin D and red cell distribution width (RDW) laboratory tests, and osteoporosis-related medication dispensations. RDW tests, although not specific to osteoporosis care, were assessed as a more generic comparison to further validate the trends observed for other outcomes.

Patients with an osteoporosis healthcare encounter were identified based on the presence of diagnostic codes for osteoporosis (733.0 × from the International Classification of Diseases, Ninth Revision, Clinical Modification (ICD-9-CM) or M80 or M81 from the International Statistical Classification of Diseases and Related Health Problems, 10th Revision, Canada (ICD-10-CA)), extracted from the Discharge Abstract Database (DAD), National Ambulatory Care Reporting System (NACRS), or Practitioner Claims datasets. Osteoporosis-related physician visits were identified based on the presence of the diagnostic code of osteoporosis from the Practitioner Claims dataset. Physician visits were stratified by general practitioner visits and specialist practitioner visits (details provided in Supplementary Table 1). Both in-person and telehealth (i.e., remote) physician visits (which were introduced in March 2020) are captured within the administrative data. BMD test volumes were identified using the Canadian Classification of Health Interventions (CCI) code 3.WZ.70 extracted from the DAD and NACRS datasets, representing a sub-set of BMD tests in the province since community-based or private clinics BMD test volumes are not included in the administrative health data. Laboratory test volumes were extracted from the Alberta Precision Laboratory (APL) dataset. Osteoporosis-related treatment prescription dispenses included oral bisphosphonates, intravenous (IV) bisphosphonates, denosumab, romosozumab, teriparatide (including biosimilars), and raloxifene. Treatments were defined using Drug Identification Numbers (DINs) from the Pharmaceutical Information Network (PIN) dataset (Supplementary Table 2). Diagnostic tests, laboratory test volumes, and treatment prescription dispenses were captured at the population level, not specific to those with osteoporosis. The Population Registry dataset was used to restrict the HCRU outcomes to individuals ≥ 50 years of age. AHS geographic zones were extracted from the DAD, NACRS, and Practitioner Claims datasets at the time of service for all other HCRU outcomes. AHS geographic zones were then collapsed into urban (Edmonton, Calgary) and rural (Central, North, South) categorizations. For an overview of the Alberta Health administrative datasets, see Supplementary Table 3.

To facilitate access to the current information from the health system, this study was conducted using “open year” administrative health data that has not undergone the additional checks and validations implemented once the system closes these datasets and prepares them for research use.

### Data analysis

Outcomes were assessed in 3-month periods to capture seasonal variation in osteoporosis-related care patterns, as well as varying public health measures in the first 6 months of the COVID-19 pandemic in Alberta (March 15, 2020 to September 14, 2020; Supplementary Figure 1). In the first 3-month period of the COVID-19 pandemic (March 15, 2020 to June 14, 2020), a Public State of Emergency was declared in Alberta, Canada, resulting in temporary lockdowns/closures and in particular, limited laboratory services [18], closure of community-based imaging services, cancelation/postponement of elective surgeries [19], restrictions around prescription days supply (e.g., limiting to 30-day supply) [20], and the introduction of telehealth visits (including ICD codes for telehealth visits) (Supplementary Figure 2).

Descriptive statistics were calculated to summarize the frequency of osteoporosis diagnostic codes and resource use and compare the change in outcome during the pandemic 3-month periods to the weighted average of the control years 3-month periods (2017/18–2019/20). In addition, outcomes were depicted visually to show increasing or decreasing trends over the study period. Treatment utilization was analyzed as the number of patients on each treatment per period based on the days supply. Treatment prescription dispenses were analyzed as treatment initiations and treatment disruptions. Treatment initiation was defined as any new osteoporosis-related prescription dispensed in each three-month period with no prior osteoporosis-related prescription dispensed in the prior 1.5 years. Treatment disruptions in each 3-month period were defined as a gap in treatment dispenses after the days’ supply plus 60 days. This definition was selected in order to measure systemic disruptions in treatment utilization. The days’ supply for oral bisphosphonates, romosozumab, teriparatide, and raloxifene was based on the days supply within the PIN dataset. For IV bisphosphonates and denosumab, the days’ supply data within PIN was not reflective of the duration of treatment effect; therefore, the days’ supply was set at 365 days for IV bisphosphonates and 182.5 days for denosumab, based on published literature [21]. The June to September COVID-19 2020 period was excluded from the treatment disruption analysis due to a lack of sufficient follow-up to assess disruptions.

Results with sample sizes < 10 were not reported to align with Alberta Health privacy standards. All analyses were conducted in SAS® version 9.4 and figures produced using Tableau online version 2021.3. Research ethics board approval was obtained from the Health Research Ethics Board of Alberta – Community Health Committee (HREBA-CHC).

## Results

### Osteoporosis-related healthcare encounters

**Fig. 1:**
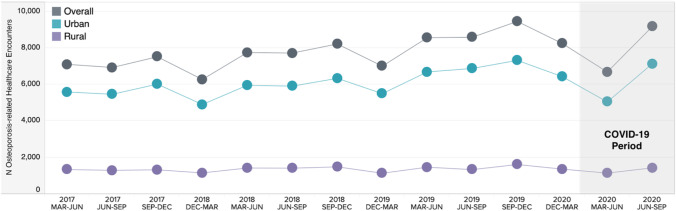
The number of patients with osteoporosis-related healthcare encounters in Alberta, Canada, by 3-month control and COVID-19 periods. Abbreviations: COVID-19, coronavirus disease 2019; Dec, December; Jun, June; Mar, March; Sep, September. Note: The urban zone was defined as Calgary or Edmonton Alberta Health Services geographic health zones, while the rural zone was defined as Central, North and South Alberta Health Services geographic health zones. Note: Osteoporosis-related healthcare encounters include visits/admissions to the hospital, ambulatory care (including emergency department and outpatient), and practitioner claims as represented by an osteoporosis diagnostic code for the visit reason

An overall slight increase in the number of osteoporosis-related healthcare encounters was observed over the study period with declines observed during the winter months, aligned with seasonal variations. However, during the initial 3-month period (March to June 2020) of the COVID-19 pandemic, large declines were also observed (Fig. 1; Table 1). When comparing the COVID-19 March to June 2020 period to the weighted average from the control March to June periods, there were 14% fewer patients with osteoporosis-related healthcare encounters in the 2020 COVID-19 period. When comparing the June to September periods, the 2020 COVID-19 period had 18% more patients than the weighted average from the control periods. When stratified by urban and rural zones, and initial decrease in the March to June 2020 COVID-19 period (− 17% vs − 19%) was observed in the number of patients with osteoporosis-related healthcare encounters following by an increase in the June to September 2020 COVID-19 period (17% vs 6%) with the inclusion of telehealth visits, relative to the weighted average control periods, respectively.

**Table 1:** Osteoporosis-related healthcare encounters, osteoporosis-related practitioner visits, BMD diagnostic test volumes, laboratory test volumes, and treatment patterns in Alberta, Canada, by weighted 3-month control and COVID-19 periods

Outcomes	March–June			June–September		
	Control (2017–2019)	COVID-19 (2020)	% Change (95% *CI*)	Control (2017–2019)	COVID-19 (2020)	% Change (95% *CI*)
*N* patients with osteoporosis-related healthcare encounters	7,805	6,683	− 14% (− 14%, − 15%)	7,744	9,166	18% (17%, 19%)
Osteoporosis practitioner visits						
General	7,211	6,266	− 13% (− 12%, − 14%)	7,293	9,018	24% (23%, 25%)
Specialist	1,095	995	− 9% (− 7%, − 11%)	1,008	1,392	38% (34%, 42%)
Diagnostic and laboratory test volumes						
BMD	1,065	562	− 47% (− 43%, − 52%)	1,068	798	− 25% (− 22%, − 28%)
Vitamin D	1,191	1,034	− 13% (− 11%, − 15%)	1,085	2,078	92% (86%, 97%)
RDW	209,849	133,490	− 36% (− 36%, − 37%)	197,459	179,639	− 9% (− 9%, − 9%)
Number of patients initiating*						
Oral bisphosphonates	3,619	2,073	− 43% (− 41%, − 45%)	3,653	3,137	− 14% (− 13%, − 15%)
IV bisphosphonates	150	111	− 26% (− 18%, − 36%)	141	183	30% (21%, 40%)
Denosumab	198	129	− 35% (− 27%, − 44%)	173	229	33% (24%, 42%)
Proportion of patients with a treatment disruption						
Oral bisphosphonates	14.8	14.8	0% (0%, 0%)	14.6	NA	NA
IV bisphosphonates	10.3	12.3	22% (2%, 79%)	9.2	NA	NA
Denosumab	8.7	11.9	41% (8%, 112%)	7.8	NA	NA

*COVID-19*, coronavirus disease 2019; *BMD*, bone mineral density; *NA*, not applicable; *NR*, not reported; *RDW*, red cell distribution width. *Patients initiating an osteoporosis treatment without having received any osteoporosis treatment in the previous 1.5 years. Note: treatment disruptions from the June to September 2020 period are not available at this time due to insufficient follow-up to identify treatment gaps of days’ supply + 60 days

### Practitioner visits, BMD tests, and laboratory test volumes

**Fig. 2:**
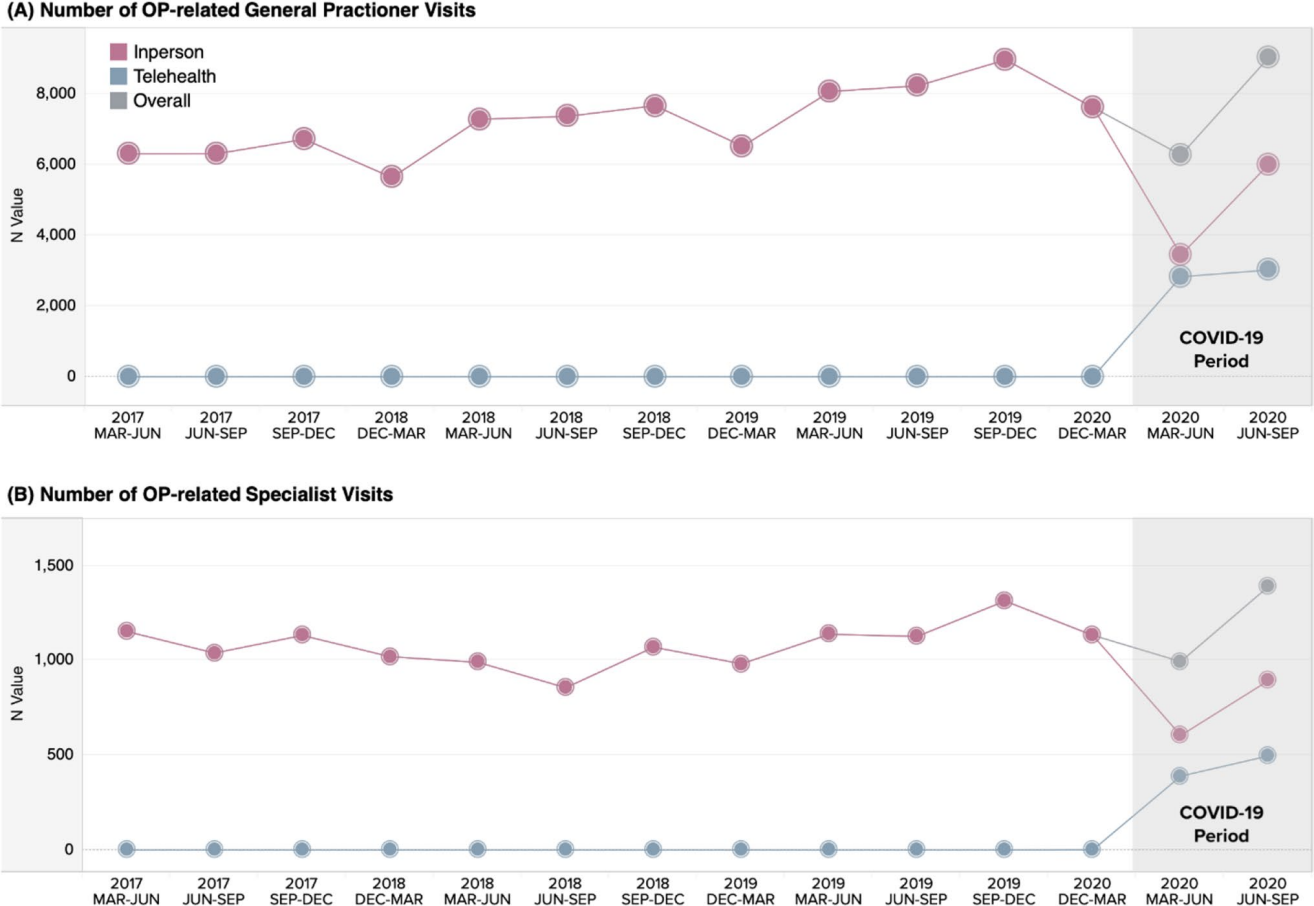
The number of osteoporosis-related general and specialist practitioner visits in Alberta, Canada, by 3-month control and COVID-19 periods. Abbreviations: COVID-19, coronavirus disease 2019; Dec, December; Jun, June; Mar, March; Sep, September

**Fig. 3:**
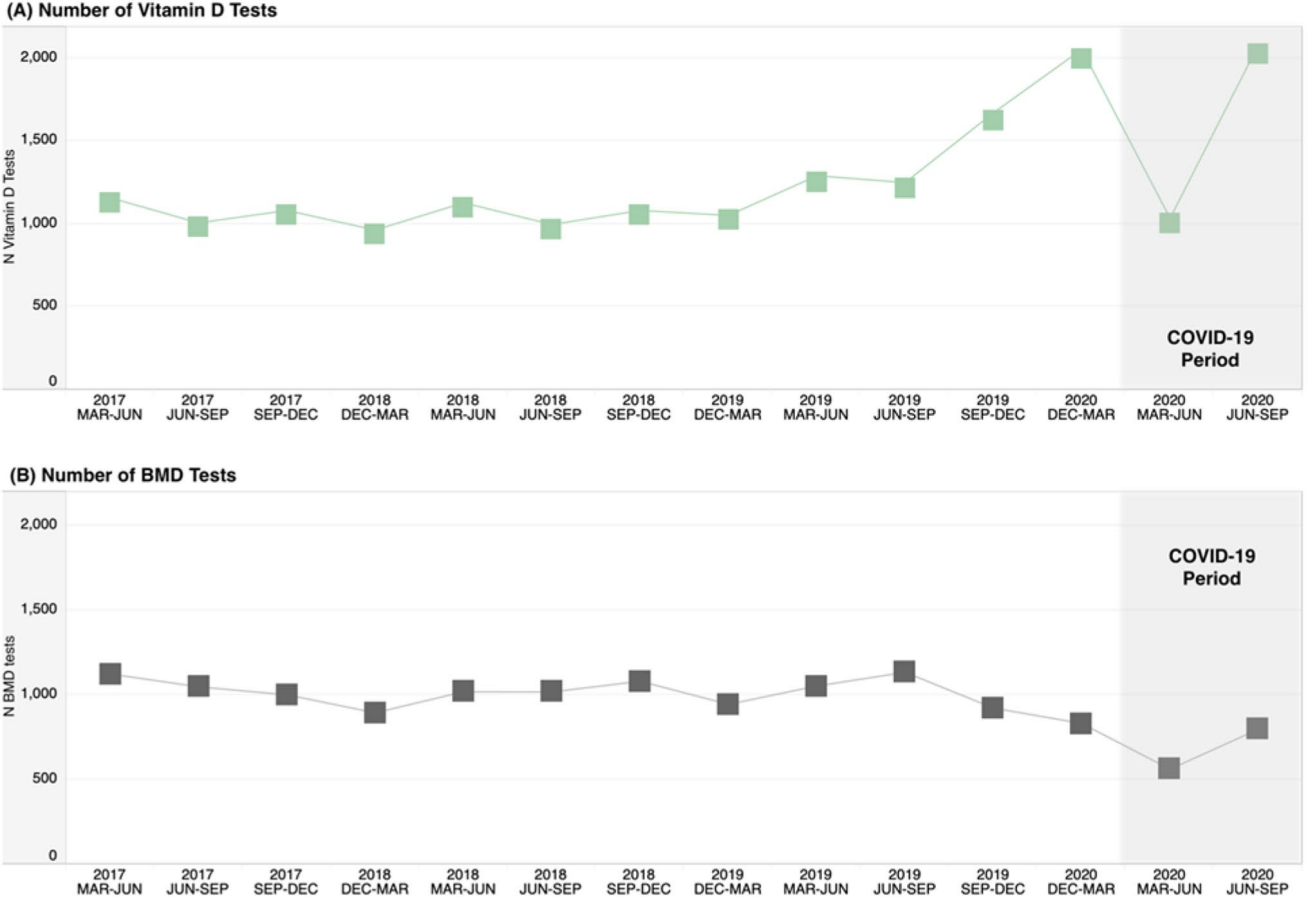
The number of vitamin D laboratory tests and BMD tests in Alberta, Canada, by 3-month control and COVID-19 periods. Abbreviations: COVID-19, coronavirus disease 2019; Dec, December; Jun, June; Mar, March; Sep, September

Reductions in HCRU for physician visits, laboratory tests, and hospital- and ambulatory care-based BMD tests were observed during the March to June 2020 COVID-19 3-month period, relative to the weighted average control period (Figs. 2 and 3 and Table 1). General practitioner and specialist visits rebounded in the June to September 2020 COVID-19 pandemic period, with 24% and 39% greater visits than observed in the same June to September pooled average control periods; however, the type of visits altered with the introduction of telehealth visits. Prior to March 2020, all visits were in person, whereas telehealth visits accounted for 2,825 (45%) and 390 (39%) of general and specialist practitioner visits in the March to June 2020 COVID-19 3-month period, respectively. Similarly, in the June to September 2020 COVID-19 3-month period, 3016 (33%) and 497 (36%) of general and specialist practitioner visits were conducted via telehealth (Fig. 2). The total number of in-person visits remained lower in June to September 2020 (GP visits = 6,002; specialist visits = 895) than in the weighted average of the control periods (GP visits = 7,293; specialist visits = 1,008).

Notable decreases of 47% were observed for BMD test volumes in the March to June 2020 COVID-19 period; however, unlike other HCRU, volumes remained 25% lower when comparing the June to September 2020 COVID-19 period to the weighted average control period. Vitamin D lab test volumes saw smaller decreases of 13% in the March to June 2020 COVID-19 period and then a substantial increase of 92% for the June to September 2020 COVID-19 period. RDW test volumes, a more generic laboratory test, followed a more similar pattern to the BMD tests, with a 35% decrease in the March to June period and remaining 9% lower in the June to September COVID-19 2020 periods (relative to the weighted average control years).

### Treatment outcomes

**Fig. 4:**
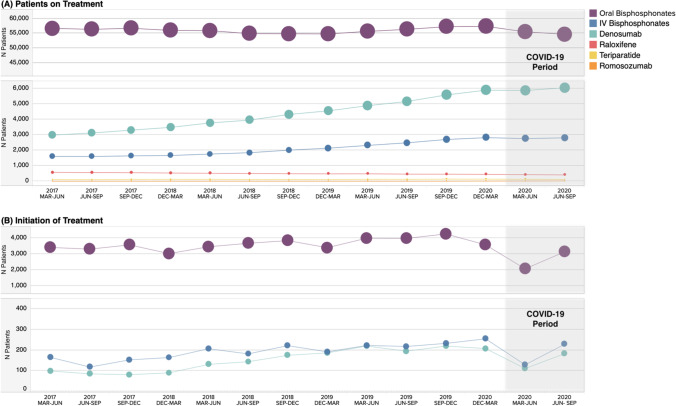
Patients on and initiating* new osteoporosis-related treatments in Alberta, Canada, by 3-month control and COVID-19 periods. Abbreviations: COVID-19, coronavirus disease 2019; Dec, December; IV, intravenous; Jun, June; Mar, March; Sep, September. *Patients initiating an osteoporosis treatment without having received any osteoporosis treatment in the previous 1.5 years. Note: the size of the dots is indicative of the number of patients

Overall utilization of osteoporosis-related medication dispenses are presented in Fig. 4. Oral bisphosphonates remained relatively stable throughout the control periods and minimal changes were observed between the control periods and the 2020 COVID-19 periods. An increasing pattern was observed in IV bisphosphonates and denosumab utilization across the control periods, as well as the 2020 COVID-19 periods. However, IV bisphosphates had a decrease in use in the initial March to June 2020 COVID-19 period, before continuing in the upward pattern.

Reduced rates of treatment initiation (i.e., initiation of an osteoporosis treatment with no prior receipt of any osteoporosis treatment in the prior 1.5 years) of oral bisphosphonates (− 43%), IV bisphosphates (− 26%), and denosumab (− 35%) were observed during the March to June 2020 COVID-19 period, relative to the weighted average of the March to June control period (Table 1). In the June to September 2020 COVID-19 period, oral bisphosphonate treatment initiations reduced by 14%, while IV bisphosphonates and denosumab treatment initiations increased by 30% and 33%, relative to the weighted average control period, respectively.

**Fig. 5:**
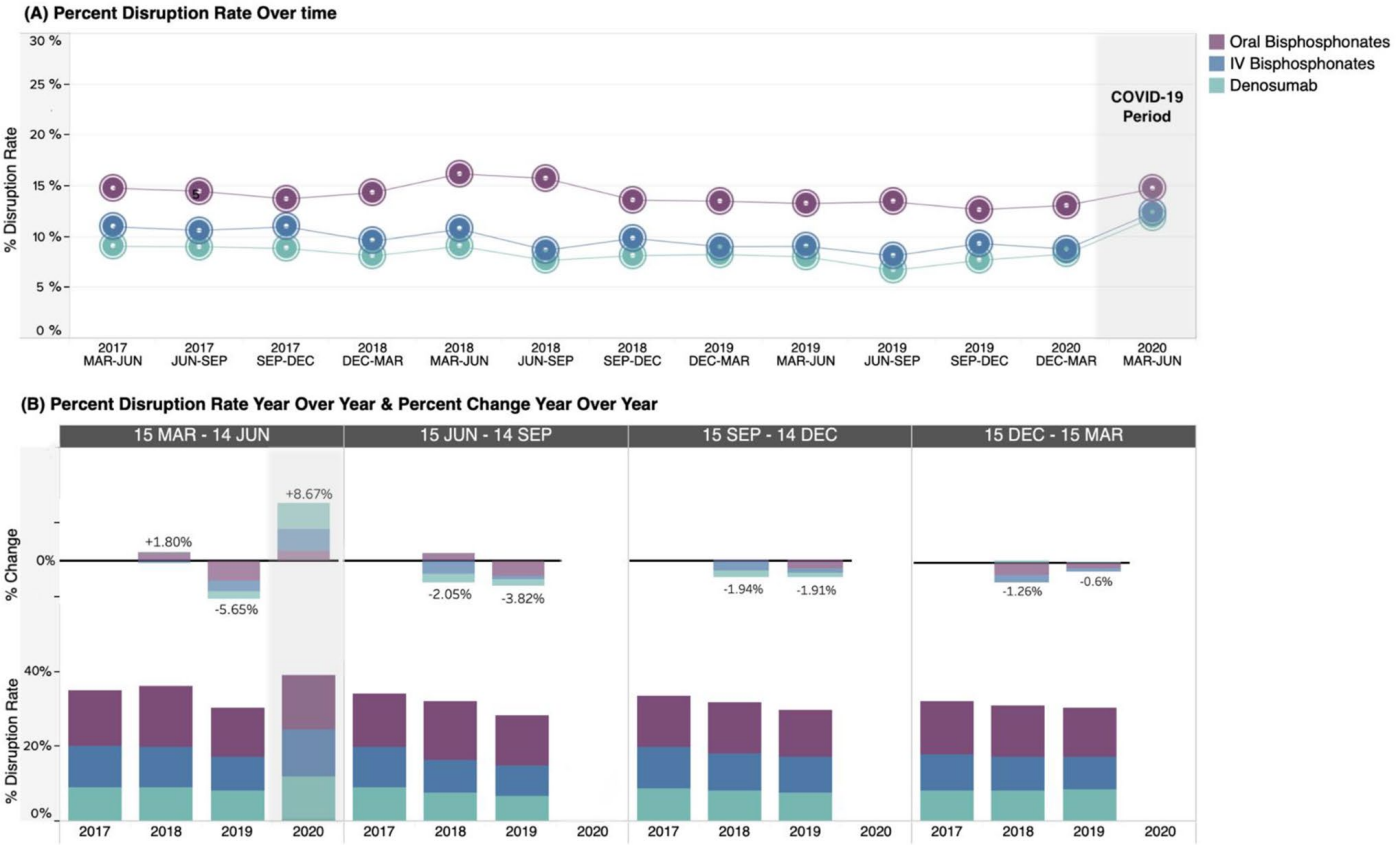
Disruptions of greater than 60 days in osteoporosis-related treatments in Alberta, Canada, by 3-month control and COVID-19 periods. Abbreviations: COVID-19, coronavirus disease 2019; Dec, December; IV, intravenous; Jun, June; Mar, March; Sep, September. Total percent change in disruption rates is presented year over year. Note: treatment disruptions from the June to September 2020 period are not available at this time due to insufficient follow-up to identify treatment gaps of days’ supply + 60 days

The proportion of patients with treatment disruptions of greater than 60 days increased slightly in the March to June 2020 COVID-19 period for IV bisphosphonates and denosumab relative to the weighted average of the March to June control period (Table 1; IV bisphosphonate: 2.1% increase; denosumab: 3.2% increase). Across the control period, the proportion of patients with treatment disruptions had a slight declining trend; therefore, when considering the percent change in the proportion of patients with treatment disruptions year over year, the March to June 2020 COVID-19 period saw a total increase of 8.7% relative to the March to June 2019 period (Fig. 5).

Romosozumab, teriparatide (including biosimilars), and raloxifene prescription initiations and disruptions had sample sizes < 10 across the three-month periods and were therefore not reported.

## Discussion

While patients with osteoporosis-related healthcare encounters have slightly increased in 3 years prior to the COVID-19 pandemic, this study observed notable reductions in the number of patients with osteoporosis-related healthcare encounters in the first 3-month period (March to June 2020) of the COVID-19 pandemic, when public health lockdowns were implemented. The COVID-19 pandemic also impacted the management of patients with osteoporosis as observed by reductions in HCRU including physician visits, hospital- and ambulatory care-based BMD tests, laboratory tests, and osteoporosis treatment initiations and disruptions.

The current study revealed a slight increasing pattern in the number of osteoporosis-related healthcare encounters reported in each period throughout the control years. While a low number of patients with osteoporosis-related healthcare encounters was observed during the March to June 2020 period of the COVID-19 pandemic when lockdown measures were implemented, this appear to rebound once lockdown measures were removed in the June to September 2020 COVID-19 period, with the inclusion of telehealth visits, accounting for 33–36% of healthcare visits. Similar patterns were observed across the study periods in the number of patients with osteoporosis-related healthcare encounters when stratified by rural versus urban region. Interestingly, this study reveals a higher number of patients with osteoporosis treatments dispenses than patients with osteoporosis-related healthcare encounters in each period. This pattern supports the evidence suggesting a lack of application to assess patients for a diagnosis of osteoporosis, which may result in the general undermanagement of the disease as a whole.

There was an overall trend observed during the study period of stable specialist visits and increasing general practitioner visits including telehealth visits, revealing a possible shift in the management of patients with osteoporosis in Alberta. As anticipated, large reductions were observed in the number of global physicians visits during the first COVID-19 pandemic period. This aligns with the changes in health care measures implemented in Alberta on March 2020 [18–20, 22]. In addition to the lockdown measures, these reductions may also be due to lack of personal protective equipment (PPE) measures in place to enable in-person visits, hesitancy for patients to visit specialists practicing within hospitals, and the practical implementation of telehealth visits. Laboratory and diagnostic test utilization were also stable across the control period with major reductions in test volumes observed during the March to June 2020 COVID-19 period. The reduction in laboratory services can be attributed to public health guidance recommending cessation of routine testing for stable community patients [18]. BMD testing remained at reduced volumes in the June to September 2020 COVID-19 period, relative to the control periods. As a note, the BMD test volumes are based on BMD tests captured in hospital and ambulatory care settings (including emergency care and outpatient visits) across the province; therefore, changes in the use of BMD testing in community-based and private clinic settings, which represents the majority of BMD testing, were not captured in this study.

The reduction in HCRU measures observed in this study aligns with reports from other regions. A survey of health care providers from 53 countries revealed numerous institutions/clinics had closures during the pandemic, or changed services to focus on emergency care only and relied on other modes of visits to facilitate care [16]. A greater proportion of physicians in this survey report using telemedicine appointments for established patients (40%) then for new patients (19%). Telemedicine was introduced as a billing option in Alberta on March 2020, creating this as an option for physician visits [22], likely diminishing the impact of the pandemic lockdowns on the levels of physician visits observed. In the first 6 months of the pandemic, telehealth accounted for 33–45% of general practitioner and 36–39% of specialist visits. The use of telemedicine may vary by physician and patient, based on comfort with technology, infrastructure, accessibility, and institutional mandates [23].

Joint guidance was released by numerous societies and organizations on the management of patients with osteoporosis during the COVID-19 pandemic [24]. These recommendations were to limit laboratory and BMD testing when possible and utilize telehealth and altered treatment approaches to reduce the need for in-person visits. These recommendations were made to facilitate social distancing practices and aligned with the Public Health measures implemented in Alberta on March 2020 [18–20, 22]. Specifically, regarding treatment, the recommendation for patients initiating osteoporosis medication was to initiate on oral bisphosphate therapy via telehealth appointments [24]. For patients currently on treatment, the recommendation was to continue their current medication. In some cases where it was not feasible to continue treatment with injectables, treatment switches were recommended. For example, patients on denosumab were recommended to switch to oral bisphosphonates if the treatment disruption would be greater than 30 days (7 months from last administration) [24]. Alternatively, for patients on IV bisphosphonates, delays of several months would be acceptable. While the number of patients initiating an osteoporosis treatment in the current study decreased in the March to June 2020 COVID-19 period for all three drugs reported, likely due to the reduced number of physician visits during this time, initiations of IV bisphosphonates and denosumab were observed to be greater in the June to September 2020 COVID-19 period, relative to the weighted average of the same control periods. However, oral bisphosphonates initiations remained lower during the June to September 2020 COVID-19 period than the average of the control periods. These initiation patterns support European estimates of reduced initiation of oral bisphosphates that continued past the initial COVID-19 pandemic lockdown periods [17], but are unexpected based on the recommendations. The proportion of patients with treatment disruptions in the first period of the COVID-19 pandemic (March to June 2020) was fairly stable relative to the weighted average from the control period. In some countries, self-administration of denosumab is not in the approved product label, likely contributing to the anticipation of disruptions, and thus motivating the treatment recommendations discussed above. However, in Canada, self-administration has been approved [25] and many patients may have already been trained in self-administration, therefore, mitigating the impact of the pandemic restrictions/lockdowns. Continued use of self-administered injectable drugs may be a strategy to help reduce treatment disruptions moving forward, outside of the pandemic, to improve patient care. Additionally, denosumab can be administered by pharmactists in Alberta precluding the need for physician visits, which may also have impacted the treatment patterns observed in this study. Lastly, many of the treatments considered in this study have long days supply; therefore, further follow-up is likely required to better assess the impact of the pandemic on osteoporosis treatment disruptions.

While lockdown measures were implemented during the first three months of the COVID-19 pandemic in Alberta, the number of COVID-19 cases and hospitalizations during this time were low. Subsequent waves of the pandemic saw much higher cases within Alberta resulting in capacity issues within the health system, warranting further analyses. Additional analyses are underway with data extended up to March 2021 to explore how osteoporosis-related clinical outcomes are associated with treatment disruptions. Additionally, longer follow-up will enable greater exploration of the trends in telehealth for patint management. The impact of shifting to telehealth on patient management and outcomes will be an important area of research to guide policy and practice patterns. Future research could also explore other osteoporosis-related laboratory tests, including serum calcium and creatinine levels. Further research focusing on these laboratory test levels in patients with osteoporosis would be valuable to examine since practitioners request these test values before initiating treatment for osteoporosis to account for any contraindications for osteoporosis-related treatments. Lastly, other areas for future research could compare regional differences in care strategies for individuals with osteoporosis and their corresponding clinical outcomes to help assess the generalizability of these findings to other regions within Canada. Such research may help inform approaches to improve the care for patients with osteoporosis and osteoporosis-related outcomes, such as fractures.

Along with future research directions, several strengths of this study should be noted. First, we used comprehensive population-based datasets, representative of the entire population of Alberta, Canada. Particularly, treatment patterns were obtained from community-based pharmacy dispensation records, which include dispensations from public, private, and out of pocket plans. Second, the repeated cross-sectional design utilized control periods three years before the COVID-19 pandemic to identify trends in the outcomes of interest. Third, examining osteoporosis-related healthcare encounters by urban and rural residence helps highlight the care gap in individuals, regardless of residency and account for variation in changes in care during the pandemic that may have occurred due to differences in COVID-19 case counts.

Although this study provided a unique opportunity to examine osteoporosis care patterns before and during the COVID-19 pandemic, there are limitations to consider when interpreting these results. First, open year data was used; therefore, the administrative data for this study had not been cleaned and validated by Alberta Health prior to analysis. Some outcomes of interest demonstrated increasing trends over the control periods; therefore, the weighted average from the control period may underestimate the percent change during the COVID-19 periods. Due to this, outcomes were also presented per period in figure format to depict the trends over time and provide a more comprehensive perspective of the potential change during the COVID-19 period. On another note, BMD test volumes are likely underestimated since our study only included BMD captured in hospital, emergency department, and outpatient care facilities. Other community-based or specialized clinics that conduct BMD testing were not captured in the administrative data. While treatment disruptions were captured during the March to June 2020 COVID-19 3-month period, reasons for these treatment disruptions are not known and only based on the follow-up data available. Therefore, it is not possible to accurately determine if patients resumed treatments after a disruption or if a disruption occurred based on long dosing intervals for some treatments (e.g., denosumab and IV bisphosphonates). Furthermore, for the treatment disruption analysis, patients in the last study period (June to September 2020) had insufficient follow-up available to meet the threshold of 60 days required to identify treatment disruptions; therefore, this data was not reported. Further analysis with extended data will help address this limitation. Lastly, the data from this study is from the province of Alberta; therefore, may not be generalizable to the rest of Canada. However, many regions within Canada imposed similar lockdown measures so these results are anticipated to provide relevant insights for such regions as to the impact on patients with osteoporosis.

## Conclusion

There was a known care gap in the management of osteoporosis prior to the COVID-19 pandemic and this evidence demonstrates the COVID-19 pandemic and corresponding public health lockdowns has further heightened this “crisis.” Understanding the impact of the COVID-19 pandemic on this patient population is fundamental to improve care and management of patients with osteoporosis moving forward. Further investigation regarding acute care, treatment patterns, and the use of telehealth throughout the course of the COVID-19 pandemic is warranted; however, this data from the initial 6 months may be beneficial for decision makers when implementing future public health measures.

## Supplementary Information

Below is the link to the electronic supplementary material.

Supplementary file1 (DOCX 125 KB)

## Data Availability

Data was obtained via data request to Alberta Health.

## Code availability

Study methodology documentation and statistical programming code used in this study is available upon request.
