# Supplementary material for: Trends in osteoporosis care patterns during the COVID-19 pandemic in Alberta, Canada
Source: Arch Osteoporos. 2022 Aug 3;17(1):110. doi: 10.1007/s11657-022-01132-7 (PMC9349101; doi:10.1007/s11657-022-01132-7)
Supplement: Supplementary file 1 — Supplementary file1 (DOCX 125 KB) [file 11657_2022_1132_MOESM1_ESM.docx]

# Supplemental Materials


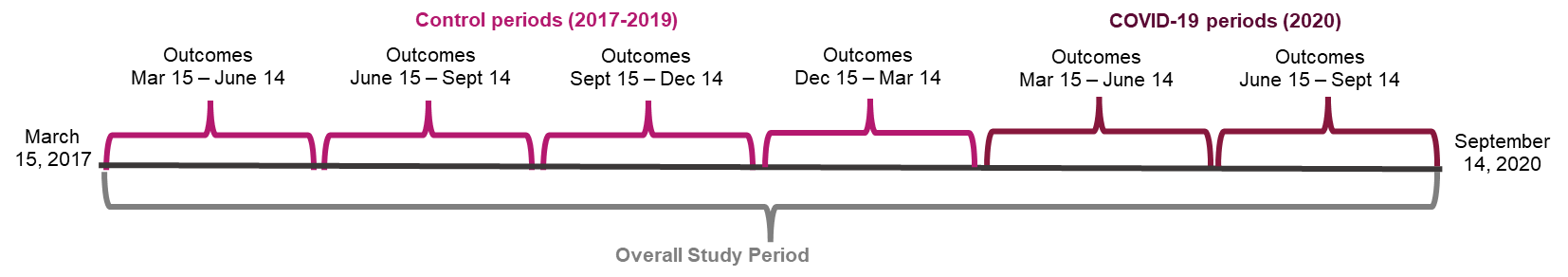


Supplementary Figure 1: Repeated cross-sectional study design

Abbreviations: COVID-19: coronavirus disease 2019; Dec: December; IV: intravenous; Mar: March; Sept: September.


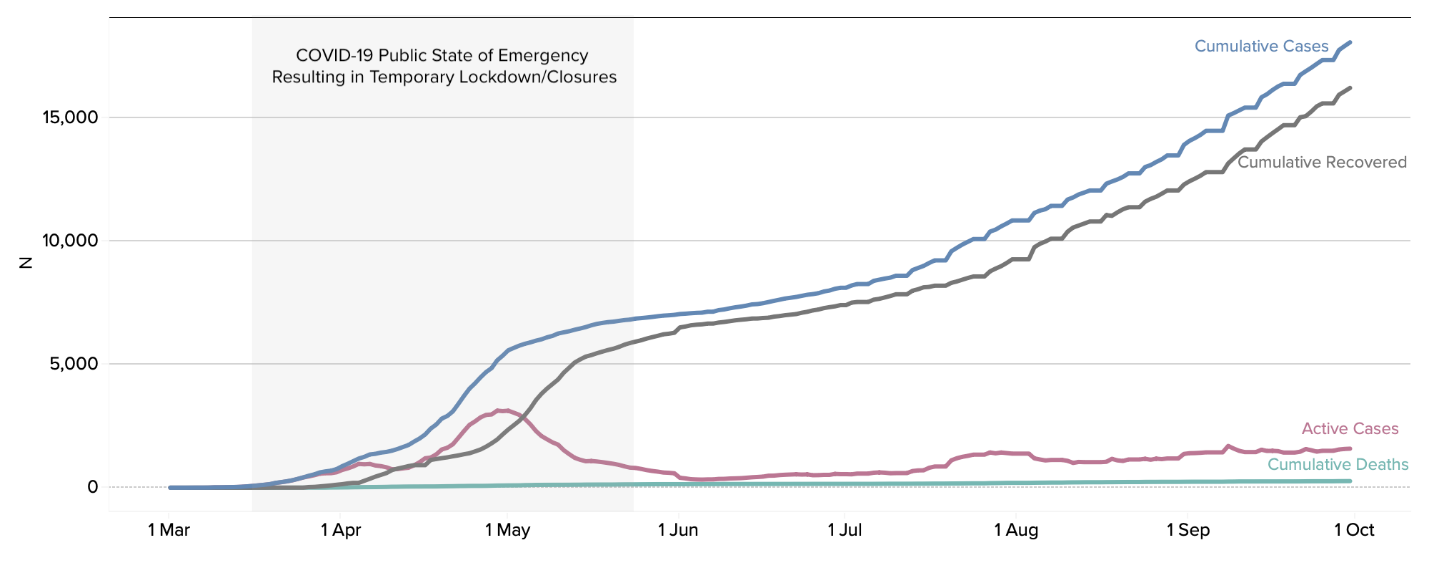


Supplementary Figure 2: COVID-19 cases across Alberta from March 1, 2020, to September 14, 2020

Abbreviations: COVID-19: coronavirus disease 2019.

Note: COVID-19 epidemiological data were sourced from the COVID-19 Canada Open Data Working Group.

Supplementary Table 1: Physician Specialties from the Practitioners Claims Database

| **Physician visit type** | **Physician specialities** |
| --- | --- |
| General practitioner | General/family physicians |
| Specialist practitioner | Anaesthesiology, cardiology, dermatology, emergency medicine, general surgery, internal medicine, neurology, obstetrics-gynaecology, ophthalmology, otolaryngology, paediatrics, pathology (laboratory medicine), physical medicine and rehab, psychiatry, radiology (diagnostic imaging), respiratory medicine, specialist surgery, urology, non-medical, all others |

Supplementary Table 2: Treatment Classifications for Osteoporosis-related Treatments

| **Drug name** | **Drug Identification Number** |
| --- | --- |
| Denosumab | 2343541 |
| Romosozumab | 2489597 |
| Bisphosphonates, oral | **Alendronate:**  2401126, 2401134, 2381478, 2381486, 2381494, 2258102, 2258110, 2299712, 2302004, 2352966, 2303078, 2248727, 2248728, 2248730, 2454467, 2454475, 2388545, 2388553, 2282763, 2308398, 2201011, 2201038, 2233055, 2245329, 2248625, 2270110, 2385031, 2394863, 2394871, 2270129, 2286335, 2282771, 2273179, 2284006, 2372304, 2384698, 2384701, 2384728, 2275279, 2270889, 2288079, 2288087, 2288109, 2429160, 2247373, 2248251, 2261715, 2403633, 2403641, 2428717, 2428725, 2428733, 2276429, 2314940, 2485168, 2485176, 2485184, 2248729, 2365057, 2365065, 2365073, 2365081, 2500175, 2385015, 2385023, 2394855, 2476398, 2476401, 2401118, 2343916, 2343924, 2405717, 2405725, 2270870, 2288095  **Etidronate:**  2263866, 2248686, 2248687, 2276844, 2276852, 2176017, 1908480, 1997629, 2347989, 2353210, 2347962, 2347970, 2247323, 2245330, 2324199, 2358697  **Risedronate:** 2239146, 2242518, 2246896, 2297787, 2316838, 2370417, 2279657, 2353687, 2377721, 2406284, 2406292, 2406306, 2442760, 2309831, 2368552, 2357984, 2397773, 2358883, 2358891, 2358905, 2427354, 2309874, 2302209, 2424177, 2362414, 2377446, 2319861, 2347474, 2352141, 2370239, 2370247, 2370255, 2411407, 2341077, 2327295, 2298376, 2298384, 2298392, 2413809, 2285541, 2326981 |
| Bisphosphonates, intravenous | **Zoledronic Acid:**  2269198, 2408449, 2408325, 2403056, 2415100, 2415186, 2426412, 2421720, 2401606, 2304007, 2424894, 2407639, 2421550, 2434458, 2444739, 2413701, 2420961, 2422425, 2408082, 2422433, 2248296, 2242725, 2482525, 2472805, 2479311 |
| Teriparatide  (including biosimilars) | 2254689, 2498804, 2495589, 2486423 |
| Raloxifene | 2358840, 2279215, 2239028, 2358921, 2415852, 2312298 |

Supplementary Table 3: Administrative Data Sources Used in Study

| **Dataset** | **Description*** |
| --- | --- |
| Ambulatory Care – National Ambulatory Care Reporting System | Includes data from all emergency department-based and community-based ambulatory care, including information on services, diagnostic and procedure codes |
| Inpatient Hospitalizations – Discharge Abstract Database | Includes data from inpatient stays, including information on services, diagnostic and procedure intervention codes as well as length of stay |
| Alberta Precision Laboratories Dataset | Includes aggregated laboratory tests volumes |
| Pharmaceutical Information Network Dispenses | Includes information on medication dispenses and associated information at the pharmacy level (all private and public plans) |
| Population Registry | Includes basic demographic information, including age, gender, and zone |
| Practitioner Claims | Includes provider claims data for physicians and other providers for insured health services, and reports on provider and service data |

*Taken from the Alberta Health, Analytics and Performance Reporting Branch – Overview of Administrative Health Datasets, April 28, 2017 - ^©^2017 Government of Alberta
